# Effects of valproate on Tax and HBZ expression in ex vivo cultured ATL cells

**DOI:** 10.1186/1742-4690-11-S1-P39

**Published:** 2014-01-07

**Authors:** Gildas Belrose, Hélène Gazon, Jean-Côme Meniane, Stéphane Olindo, Jean-Michel Mesnard, Jean-Marie Péloponèse, Raymond Césaire

**Affiliations:** 1 Laboratoire de Virologie and EA 4537, Centre Hospitalier Universitaire de Fort-de-France, Martinique, France; 2 Centre d'Études d'Agents Pathogènes et Biotechnologies pour la Santé, CNRS UMR 5236, Université Montpellier 1, Université Montpellier 2, Montpellier, France; 3 Service d’Hématologie clinique, Centre Hospitalier Universitaire de Fort-de-France, Martinique, France; 4 Service de Neurologie, Centre Hospitalier Universitaire de Fort-de-France, Martinique, France

## 

Epigenetic drugs are known to regulate expression of tumor suppressor genes and activities of transcriptional factors involved in both cancer initiation and progression. Valproate (VPA) has been shown to enhance Tax expression and block HBZ-mRNA expression in HTLV-1 and HAM/TSP lymphocytes. HBZ is critical for immune escape and proliferation of ATL cells. We evaluated the impact of VPA on Tax, Gag and HBZ expression in ex vivo cultured ATL cells. Samples were obtained from 6 patients with acute ATL, 4 patients with HAM/TSP, and 3 asymptomatic carriers (AC). CD8+-cell-depleted PBMCs were cultured during a week with or without 5mM VPA. HTLV-1 mean proviral loads at day 0 of culture were 58 copies/100 cells, 7 copies/100 cells, and 4 copies/100 cells in ATL, HAM/TSP, and AC, respectively. At day 5, mean proviral loads decrease by 2 fold in VPA treated vs. untreated ATL samples. Mean±SD apoptosis at day 1 was 11%±6 in untreated versus 23%±11 in VPA-treated non-ATL samples, and 13%±9 in untreated versus 43%±25 in VPA-treated samples. Tax expression in CD4+ cells, measured by Facs and by qRT-PCR, peaked at day 1 of culture in AC and HAM/TSP samples but not in ATL samples. Tax and gag mRNAs were undetectable during culture of ATL untreated samples, whereas HBZ was expressed. VPA treatment of ATL samples significantly increased Tax and Gag mRNA mean levels, and blocked HBZ mRNA expression. The possibility to target HBZ expression using VPA at therapeutically useful concentrations opens a new way for the treatment of ATL.

